# Income-Tax Liabilities and Claims

**Published:** 1919-08-30

**Authors:** John Burns

**Affiliations:** Edinburgh


### INCOME-TAX LIABILITIES AND CLAIMS.

By JOHN BURNS, W.S., EDINBURGH.

It can scarcely be affirmed that either the generalities or the details of income-tax matters are simple affairs from the taxpayer's point of view, and undoubtedly to the busy practitioner there is a very considerable risk of going beyond his duty to his country and so far neglecting his duty to himself and his family by paying an amount of tax beyond his true liability. Putting it as briefly as possible, the things to be kept in view are to see—

1. That you do not send in, or submit to, an over-estimate of net professional profits.

2. That you do not commit the mistake of treating as taxable income what the law does not so regard.

3. That you deduct all "legal burdens" such as ground charges, interest, annuities.

4. That you obtain all reliefs and abatements to which you are entitled.

5. That you do not pay at a higher rate than you are liable for. This applies particularly to income from investments taxed "at the source"—that is, before you receive it—at the full standard rate. This may require a repayment claim, as, indeed, so may some of the other matters referred to.

6. That in partnership cases the income-tax charged on the firm is borne by the partners in their proper proportions. Though two partners are equally interested in profits, it does not follow, and is almost certainly not the case, that each should bear one-half of the firm's tax.

The above short paragraphs may serve as, in some sort, the heads of the sermon, but a good deal of flesh requires to be put on these dry bones before much effectual practical benefit can be obtained by those who are not experts.

### INCOME.

*Professional Income.*—Whether it is the case of an individual or a firm, the taxable income is arrived at on the average of the three preceding completed years. Note that this does not mean the amounts of the gross income-tax assessments for those years. No; it means the profits brought out in each of these years according to proper accounts made out at the end of each year. It follows that the average sum thus brought out is only an estimate, which may be more or less than the year of taxation actually yields in the way of profit, and that is quite recognised; it is called the "statutory" income. You may in making-up accounts go on either the cash basis or the earnings basis. The former pays no heed to fees earned and expenses incurred if not actually received or paid; of course these all come into account in a subsequent year. The earnings system puts down everything earned or incurred, though not yet materialised either way. In this case any errors are rectified in the later year in which the items are received or paid. The Department is indifferent which of those two methods is followed, but it is not permissible to change about from the one to the other.

In any case gross drawings are not profits; all proper expenses must be deducted. They include—rent or annual value of premises used exclusively for practice; proper share of rent or annual value of premises where the practitioner resides according to the part used for practice, not exceeding two-thirds; rent or annual value of garage or stable; inhabited house duty and tenant's rates, heating and lighting corresponding to the above; assistants and *locum tenens*; service applicable to the practice, including coachman, chauffeur, maid-servant, also secretary or book-keeper; travelling, including railway, car, carriage (not the original cost of car, carriage, or horses, for these are regarded as capital), but all running expenses, repairs, and renewals less salvage; instruments, medicines; professional subscriptions and literature; cost of collection of accounts; telephone, telegrams, stationery, and postage; bad and doubtful debts, but not if the cash system of accounts is followed, for then you enter only the actual fees received.

The three years' average rule is subject to optional modification in favour of the taxpayer in the case of new practices, discontinued practices, death, succession, purchase, partnership changes, war service.

*Public Appointments.*—This special form of professional income is not taxed on the average but on the actual income of the year. If the appointment carries an official residence, light, fuel, service, board, etc., these things—though no doubt very valuable—are not income for taxation purposes, and in your statement you ignore them altogether.

*Investment Income.*—This must, of course, go down in the statement or return. It may have been received by you under deduction of tax or without that deduction. In either case you enter the gross sum. Far more of this kind of income is now received gross than formerly was usual, this being due to the fact that interest on war loans and bonds (if not bearer) is mostly so paid. Note if the interest is received in gross, the proper amount to be put down in your return is *last* year's figure, which, of course, may sometimes be *nil*. Also pay particular attention to dividends which bear that they are "free of tax." This is only a form of expression. What it means is that if at present you receive a dividend of £14 "free of tax" the company is actually paying a gross dividend of £20, which leaves the net £14 after 6s. per £ is deducted for income-tax. Therefore you will put down £20 as income from that source; but then remember that on this full £20 you have borne 6s. per £, which may be a good deal more than your true unearned rate according to your total income. That means a repayment or adjustment claim in your favour. Finally, in the case of real estate you enter, not the gross rent, but only the net Schedule A assessment. This applies also to your residence if it belongs to yourself or your wife, for it is "income."

*Wife's Income.*—This must be massed with the husband's. The only exceptions are when the spouses are not living together; or if the lady is separately *earning* an income, and if the total income of both does not exceed £500; but, even then, her unearned income is treated as her husband's for taxation.

### DEDUCTIONS.

Suppose the figures are—Professional income (three years' average) £700; public appointments, £200; from investments, £100; wife's income, £100. That gives a total of £1,100. But then it may be that there are payable ground burdens on house, £20; mortgage interest, £80; annuity to late partner's widow, £200. These deductions amount to £300, thus reducing the income to £800, which is the practitioner's income for taxation purposes. That means that his right to abatements and his rates (earned and unearned) of tax are regulated by the £800 standard. But then he will, all the same, be taxed on

**Income-Tax Liabilities and Claims—(*continued*).**

the £300 of deductions also, and not only so, but at the full 6s. rate. The reason for that is that when he makes the payment of the ground burden, mortgage interest, and annuity, he deducts tax at 6s. So he pays not £300, but only £210. This leaves him with £90 in hand, which just enables him to pay the Revenue's demand on that part of the account, so he loses nothing.

*Bank Interest.*—But then it may be that the £80 is interest, not on a mortgage, but to a bank. That is a different case altogether, for banks are in the habit of charging interest in full, without allowing any tax deduction. In one way or another the practitioner must see that he obtains an allowance of the tax on the interest. One way is to have some of his direct assessments (say on his War Loan dividends) reduced or cancelled. Another way is to send in, once a year, a direct claim for repayment by the Department. This is a very important point, for the amount involved is often large. We should think it very likely that large sums of tax are lost in this way every year. Indeed we should not be surprised if not a few practitioners, who may happen to have bankers' loans, could not tell whether the interest is charged with, or without, deduction of tax.

